# Ethical Relationship of the Medical Profession and Life Insurance Companies

**Published:** 1894-04

**Authors:** S. H. Stout

**Affiliations:** Dallas, Texas


### TEXAS MEDICAL JOURNAL.

ESTABLISHED JULY, 1885.

PUBLISHED MONTHLY.—SUBSCRIPTION $2.00 A YEAR.

VOL. IX. AUSTIN, APRIL, 1894. NO. 10.

### Original Contributions.

For Texas Medical Journal.

### ETHICAL RELATIONSHIP OF THE MEDICAL PROFESSION AND LIFE INSURANCE COMPANIES.

BY S. H. STOUT, A. M., M. D., LL. D., DALLAS, TEXAS.

FOR more than a decade, the writer has declined to examine applicants for life insurance. He has carefully studied the subject in all its features; did in former years make many examinations of applicants, and never had a risk he recommended rejected.

He arrived at the conclusion to decline to make examinations, after studying his duty in the relationship of a medical practitioner to life insurance corporations.

It is the object of this paper to state, as briefly as possible, the facts and reasons which led to this conclusion.

Life insurance, as practiced, is a financial venture on the part of the insurer and the insured. The premium paid by the insured is calculated from data obtained from carefully studied tables of mortality. The physical, mental and moral condition of the insured mnst be normal, and his occupation and fortune, as well as the salubrity of the locality in which he lives, and his inherited diathesis, must be considered by the medical examiner before announcing his insurability, or non-insurability, to the company from which it is proposed to seek a policy.

Among a thousand individuals of the above characteristics, the tables of mortality show there is a law that every year there is an average death rate. The mathematical doctrine of chances is also raised in the calculation of premiums.

A life insurance risk is therefore, in a certain sense, a bet. The insured bets he will die before he reaches his expectation of life, when the premiums paid by him, with compound interest at 4 per cent., will equal the face of the policy; and the company issuing the policy bets that he will live out his expectancy of life indicated by the tables of mortality. Therefore the insured, if he dies before the expiring of his expectation, wins the bet, and the beneficiary, or benefits, received on his policy, the premiums paid, plus 4 per cent. compound interest for the full term of the expectation of life of the insured. If he lives out his expectation of life, or beyond it, or lives until the maturity of an endowment, or the termination of a tontine, the company issuing the policy wins the bet, and if its finances are well managed, makes a very large profit therefrom. For the money invested by the insured in the form of premiums, can be made to yield more than 4 per cent. compound interest. Again, the net premiums, calculated by actuaries, are always loaded to meet the expenses of the business of life insurance, among the largest of which are the commissions of soliciting and managing agents. These are often very large, sometimes being from fifty to seventy-five and eighty per cent. upon the first premiums paid. Then the usually enormous expenses of the administration of the business of a life insurance company, at the home office, have to be met. The salaries of the president, secretary, treasurer, the chief medical examiner and actuary, are usually excessive compared with the income of the most skilled and competent local medical examiners. Attorney's fees, clerk hire, etc., etc., are to be added to these expenses. The offices of the most successful life insurance companies are in palatial edifices, extravagantly furnished.

Capitalists make large investments in life insurance companies, whether stock or mutual, which, if they survive to a certain condition of success, are very profitable, and make large dividends both directly and indirectly.

Great financial centers seek to have the administration of these companies in their midst. For the enormous accumulation of money to be loaned on interest, by life insurance companies holding and manipulating the trust fund deposits, from which, and the compound interest thereon, policies are to be paid at maturity, is not a small factor in the accumulation of financial power.

Think of it. The aggregated amount of the life insurance policies now in force in the United States is greater than the national debt. To meet these policies, the trust fund deposits now in the hands of the companies, must be so manipulated as to amount to the face of every policy when due.

There are two sources of weakness inherent in every life insurance company, and these are the taking of risks upon impaired or uninsurable lives, and the dishonesty, or want of financial skill of the directors of the company in whose hands is the keeping and safe and profitable manipulation of the trust fund deposits.

To guard against the misuse of these funds, the several States have life insurance laws and penalties for the violation of them. In some of them there is a life insurance bureau with a competent commissioner at its head, to whom, at stated intervals, every company doing business in the State is compelled to report its operations and financial condition.

But to guard against the insuring of impaired lives (that is the taking of uninsurable risks) the practice of these great financial corporations is erroneous, for it is irrational and unbusiness like. Under it many llfe insurance companies now in existence would have been wrecked long since, but for the honesty and skill of the members of our noble and benevolent profession who have examined applicants for them, and accepted therefor fees contemptibly small, when the skill required the amounts of money at risk, and the embarrassment of the examiner when he finds that he has to report unfavorably in regard to the application of a friend and neighbor, and mayhap a patron, are considered.

The mission of medical practice is to prolong human life and ameliorate human suffering. It is this mission that dignifies it as a profession, the pursuit of which money should be the means of encouraging. It is not a mere trade, the object of which is money-getting.

Life insurance companies, therefore, have no claim upon the members of the medical profession on the score of humanity. Both the life insurance company and the applicant for life insurance propose to enter upon a money venture. The skill of the medical examiner is not sought by them before entering into the proposed contract, for the sake of prolonging life or ameliorating human suffering. The immediate medical examiner of an applicant is therefore entitled to a fee somewhat in proportion to the money at risk, and should not be called upon to accept a fee disproportionate thereto, because of any pretense of a humane consideration as a plea in abatement thereof.

The time and money expended by an insurance actuary, in acquiring a knowledge of his profession, need not amount to one tithe of the time and money necessary to prepare a physician to become an expert and reliable medical examiner of life insurance applicants.

The vast railroad properties of Jay Gould's heirs, are of less value than the sums of money now at risk in life insurance policies. Those properties give employment to thousands of men. They are therefore engaged in as much benevolent enterprises as are life insurance companies. Yet their owners do not ask professional civil engineers to give opinions and advice about the location of their railroad tracks, the construction of their buildings, bridges, and rolling stock, for fees as contemptibly small, as those life insurance companies pay their local medical examiners.

Life insurance companies pay their chief examiners enormous salaries, for coming to the office daily, spending a few hours in reading over the papers of applicants, and giving their opinion of the insurability of the risks proposed, about which they would necessarily be in profound ignorance, but for the local medical examiners' reliable reports.

Is it not time that the honorable members of the medical profession refuse to give gratuitous advice, as to the qualifications of each other, to great moneyed corporations, and examine applicants for life insurance for the fees for a long time usual and very meager conceded heretofore, under the specious claim set up by great moneyed corporations and their agents, that they are engaged in a work of pure benevolence?

An affirmative answer to this question is inevitable, in consideration, 1st, of the fact that the seeking and issuing of a life insurance policy is no more nor less than a financial venture. Inasmuch as lawyers, called upon to investigate titles to land, write mortigages, deeds of trust, or wills, or to advise executors and administrators of estates, charge and are allowed fees proportioned to the money at risk, medical examiners, are justly entitled to fees somewhat proportioned to the amounts at risk on life insurance policies.

Life insurance companies should for their own security concede fees for medical examinations thus proportioned, or appoint local medical examiners, with salaries adequate to compensate first-class medical men for their services, as do railroad corporations their local civil engineers. They should cease to impose upon the medical profession, by the false pretence, that they are purely benevolent institutions, and medical men, if they will study the subject of life insurance in all its aspects, will, as the writer has done for ten years past, refuse to be longer "sponged" upon by them or their agents, and to continue to lend medical skill for an inadequate compensation, to protect purely financial institutions, handling, in many cases, millions of money, from insolvency, and the beneficiaries of life insurance policies from loss of the investments made by the insured in their behalf.

Having thus ventilated some of the facts and considerations which have influenced his conduct in the premises, the writer does not propose to question the conscientious actions of his medical brethren, who may differ with him in opinion and practice. He believes there is, and can be, in the proper sense of the expression, no professional ethical relationship between life insurance companies and their medical examiners. For their relationship is a strictly business one.

If life insurance companies choose to do so, there is no ethical consideration that forbids them from letting out contracts for medical examination to the lowest bidders; nor is there any ethical consideration forbidding a medical examiner from making any contract he pleases with a life insurance company. Were this view of their relationship taken by the most skillful members of the medical profession, life insurance companies would no longer impose upon their benevolent instincts, and they would have to adopt the practice of paying fees to first-class medical men, for the examination of applicants, proportioned to the amount of money at risk, on every policy issued.

Good and well meaning medical men may, without a violation of medical ethics, continue to serve life insurance companies under the present system, for every one serving a business house has a right to accept as much or little wages as he chooses; but the writer does not choose to serve great moneyed corporations engaged in the business of life insurance for the pay they now allow, because he thinks his skill is worth more to them, and because (to use a Texas boy's slang) he "don't have to."

---

**To detach** a fish bone from the throat, swallow a raw egg as quickly as it can be obtained.

